# Hepcidin: A Critical Regulator of Iron Metabolism during Hypoxia

**DOI:** 10.1155/2011/510304

**Published:** 2011-09-06

**Authors:** Korry J. Hintze, James P. McClung

**Affiliations:** ^1^Department of Nutrition, Dietetics & Food Sciences, Utah State University, Logan, UT 84322, USA; ^2^Military Nutrition Division, US Army Research Institute of Environmental Medicine (USARIEM), Natick, MA 01760, USA

## Abstract

Iron status affects cognitive and physical performance in humans. Recent evidence indicates that iron balance is a tightly regulated process affected by a series of factors other than diet, to include hypoxia. Hypoxia has profound effects on iron absorption and results in increased iron acquisition and erythropoiesis when humans move from sea level to altitude. The effects of hypoxia on iron balance have been attributed to hepcidin, a central regulator of iron homeostasis. This paper will focus on the molecular mechanisms by which hypoxia affects hepcidin expression, to include a review of the hypoxia inducible factor (HIF)/hypoxia response element (HRE) system, as well as recent evidence indicating that localized adipose hypoxia due to obesity may affect hepcidin signaling and organismal iron metabolism.

## 1. Iron: An Essential Trace Element

Despite the initial identification of iron as a major component of human blood over two hundred years ago, poor iron status continues to affect billions of people worldwide. Although the prevalence of iron deficiency (diminished iron stores, ID) and iron deficiency anemia (diminished iron stores coupled with reduced hemoglobin, IDA) is greatest in the developing world, poor iron status also affects a significant portion of the population in developed nations. For example, ID and IDA affect up to 12 and 4% of premenopausal women in the United States, respectively [1]. Iron functions biochemically through incorporation into a series of proteins and enzymes. Many of these proteins and enzymes, including myoglobin, cytochrome c, and hemoglobin, are required for optimal cognitive and physical performance in humans [2–4]. Although diminished work capacity due to reduced hemoglobin levels is the best described functional consequence of poor iron status, other outcomes include diminished intellectual performance, altered body temperature regulation, and reduced immunity and resistance to infections [5]. 

**Figure 1:**
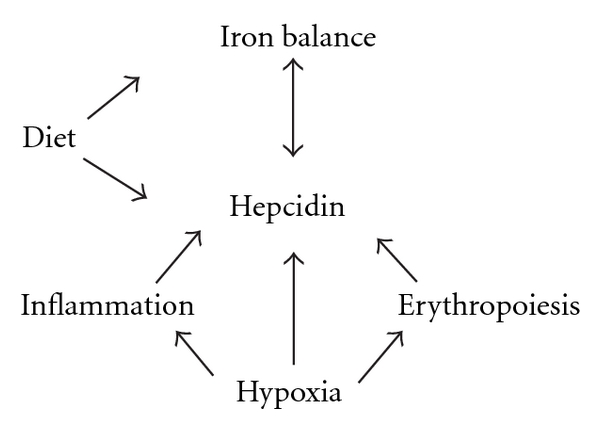
Factors affecting iron balance.

Iron balance has been historically defined as the net difference between the amount of iron that is absorbed from the diet and the amount of iron that is excreted. As such, countless investigations have focused on dietary factors affecting iron balance, such as the quantity of iron found in particular food sources and factors that may promote or inhibit iron absorption. Although iron consumption through the diet is clearly a major determinant of iron status, it has become increasingly apparent that many factors affect iron balance (Figure 1). In fact, iron balance is tightly regulated through a series of cellular transport proteins that are sensitive to the recently discovered iron regulatory hormone, hepcidin. The discovery of hepcidin, a 25-amino acid protein, has significantly altered the research paradigm in the field of iron homeostasis. Although the contribution of dietary sources to iron balance should not be ignored, hepcidin has been described as the “central regulator of iron homoeostasis,” and affects both iron absorption through the duodenal enterocyte and iron export from the macrophage and other iron exporting cells. Further, a series of factors, some of which are not directly associated with dietary iron intake, affect hepcidin levels, such as inflammation, erythropoiesis, and hypoxia. This paper will detail the mechanism by which hepcidin affects iron metabolism, with a focus on hypoxia, a factor affecting the molecular regulation of hepcidin, and, ultimately, iron status. 

## 2. Hepcidin: The Central Regulator of Iron Homeostasis

Hepcidin was first identified as an antimicrobial peptide synthesized by the liver in 2000 [6, 7]. Although hepcidin confers antimicrobial properties *in vitro,* the estimated concentrations found in biological fluids may not support a major role for this peptide hormone as an antimicrobial agent *in vivo* [8]. In 2001, the observation that hepcidin was overexpressed in response to dietary iron overload led to the hypothesis that hepcidin may function as a central regulator of iron homeostasis [9]. Subsequently, roles for hepcidin in both the acquisition of iron from the gastrointestinal tract and the efflux of iron from macrophages have been characterized.

Hepcidin regulates iron homeostasis mainly through its ability to bind ferroportin 1 (FPN1), the iron exporter responsible for iron egress from duodenal enterocytes, macrophages, and other iron exporting cells. Specifically, hepcidin binds to FPN1 on the cell surface, triggering the internalization of the hepcidin-FPN1 complex, which results in the subsequent ubiquitination of the FPN1 and the degradation of the protein complex [10, 11]. Experimental evidence confirms this mechanism of hepcidin action: disruption of the hepcidin gene leads to persistent FPN1 expression on the cell membrane and subsequent iron overload [12, 13], and delivery of synthetic hepcidin results in decreased serum iron in response to diminished intestinal iron absorption [14]. In sum, hepcidin dictates iron balance by restricting iron absorption and macrophage iron release, thereby reducing iron stores and affecting the availability of iron for conferring critical biological functions, including erythropoiesis [11]. 

Adding to the complexity of the maintenance of iron homeostasis, hepcidin synthesis is regulated by a number of factors, including iron status, inflammation, and erythropoiesis. The regulation of hepcidin by iron status appears to occur through the bone morphogenic protein (BMP) pathway. A number of BMPs, including BMP6, have been demonstrated to affect hepcidin production, *in vivo* [15–17]. For example, knockout of BMP6 in a mouse model results in severe iron overload [16, 18], and BMP6 expression increases with iron loading in mice [19]. The regulation of hepcidin synthesis in response to inflammation occurs in response to proinflammatory cytokines, including interleukin-6 (IL-6). Increased circulating concentrations of IL-6, which may occur in response to inflammatory condition such as sepsis, inflammatory bowel disease, and obesity, stimulate hepcidin transcription through a STAT3-dependent mechanism [20–24]. Associations between iron status, inflammatory biomarkers, and hepcidin levels have been documented in recent human trials [25]. Erythropoiesis, the biologic process requiring the greatest concentrations of body iron, is a suppressor of hepcidin production [24]. Both human and animal studies have demonstrated reductions in serum hepcidin and mRNA levels, respectively, following erythropoietin administration [26, 27]. Although the finite mechanism by which erythropoiesis affects hepcidin production remains unknown, early evidence indicates that two proteins produced by erythroid precursors are involved in hepcidin regulation. Growth differentiation factor 15 (GDF15) has been demonstrated to suppress hepcidin expression and is elevated in patients with thalassemia [28]. Another erythrokine, twisted gastrulation protein (TWSG1), has also been shown to suppress hepcidin mRNA *in vitro *[29]. 

Hypoxia is another factor that may affect hepcidin regulation, potentially through increased erythropoietin (EPO) production. The remainder of this paper will focus on potential mechanisms by which hypoxia may affect hepcidin levels, and, subsequently, iron homeostasis. 

## 3. Hypoxia and Iron Metabolism

The profound effects of hypoxia on organismal iron metabolism have been well described. Early studies demonstrated that hypoxia affected dietary iron absorption [30–32] and increased erythropoiesis when humans were moved from sea level to high altitude [33]. As the partial pressure of O_2_ decreases through increased elevation, anemia, or localized tissue hypoxia, a battery of genes are induced by the hypoxia inducible factor (HIF)/hypoxia response element (HRE) system. The HIF system senses O_2_ levels through degradation of HIF transcription factors (HIF-1*α* and HIF-2*α*) that are mediated by the partial pressure of O_2_ and iron-dependent hydroxylases. At normoxic O_2_ tensions, HIF-1*α* is hydroxylated by prolyl hydroxylase and then bound by the von Hippel-Lindau (VHL) protein leading ultimately to ubiquitination and proteasome degradation. During hypoxic conditions, the activity of hydroxylases is inhibited allowing HIF-1*α* to accumulate and bind along with HIF-1*β* to HRE found in the promoters of target genes. Similar to HIF-1*α*, HIF-2*α* stability is mediated by the partial pressure of O_2_ through prolyl hydroxylase and plays a dominant role in hypoxic signaling of EPO expression [34]. 

## 4. Hypoxia and Hepcidin Expression

Reduced expression of hepcidin during hypoxia was first reported in 2002 by Nicolas et al. [35], who demonstrated a marked reduction in hepcidin mRNA in HepG2 cells cultured at 2 and 0.1% O_2_ compared to standard 20% O_2_ culture conditions. Similarly, when mice were placed in atmospheric chambers emulating an altitude of 5,500 m, hepcidin expression was downregulated after 48 hrs of hypoxia. Leung et al. [36] also reported decreased hepcidin mRNA expression in HepG2 cells cultured at 1% O_2_ and in liver from rats placed in atmospheric chambers containing 10% O_2_. However, the molecular mechanisms linking hypoxia and decreased hepcidin expression remained unexplained. Because the HIF signaling system imparts a dominant role in sensing O_2_ deprivation and subsequent gene expression, including the upregulation of EPO [37], it is a probable, direct or upstream, mediator of hypoxia-induced, hepcidin gene regulation.

Peyssonnaux and colleagues [38] demonstrated that the expected hepcidin downregulation in response to dietary iron deficiency was partially blunted in hepatocyte-specific HIF-1*α* knockout mice as compared to wild-type animals, suggesting that HIF-1*α* might be a negative regulator of hepcidin signaling. Moreover, hepatocyte-specific VHL knockout mice have higher HIF-1 levels but reduced hepcidin expression and increased hepatic ferroportin levels, further suggesting a role for the HIF system as a negative hepcidin regulator. Peyssonnaux et al. [38] went on to demonstrate that murine and human hepcidin promoters contained a consensus HRE that could be immunoprecipitated by a HIF-1*α* antibody when cells were treated with the iron chelator and HIF-1*α* stabilizer desferrioxamine (DFO). Taken together, these data provided evidence that the HIF system is a negative regulator of hepcidin expression and provided a mechanism whereby hypoxia may decrease hepcidin expression. Typically, the HIF system functions as a positive regulator of gene expression [37]; however, negative transcriptional regulation by the HIF system is not unprecedented [39].

Studies by Braliou et al. [40] tested whether 2-oxoglutarate-dependent oxygenases mediated hepcidin regulation in response to hypoxia. Similar to studies by Nicolas et al. [35] and Leung et al. [36], hypoxia decreased hepcidin expression in both HepG2 and Huh7 cells. Hepcidin expression was also decreased by hypoxia mimetics, including dimethyloxaloylglycine (DMOG), DFO, and CoCl_2_. These mimetics function by stabilizing the HIF pathway by inhibiting 2-oxoglutarate-dependent oxygenases and thus preventing HIF-1*α* hydroxylation and destruction. While hypoxia, DFO, and CoCl_2_ only modestly decreased hepcidin expression, DMOG dramatically decreased hepcidin expression in both cell lines. Despite negative regulation by hypoxia and hypoxia mimetics, overexpression or knockdown of HIF-1*α* had no effect on hepcidin promoter activity suggesting that HIF-1*α* is not directly involved in hepcidin promoter transcriptional regulation. In a similar study by Choi et al. [41], hypoxia decreased hepcidin expression in both mice and HepG2 cells, and the cell culture studies indicated that decreased hepcidin expression was independent of HIF-1*α*. The authors concluded that hypoxia-induced oxidative stress decreased STAT3 and C/EBP*α* protein/DNA interactions, thus resulting in decreased hepcidin expression.

Interestingly, not all studies have demonstrated that hypoxia directly decreases *in vitro* hepcidin expression. In an investigation by Volke et al. [42], HepG2 and Huh7 cells cultured under hypoxic conditions did not have diminished hepcidin expression, contrary to previous reports [35, 36, 40]. However, similar to the study by Braliou et al. [40], the hypoxia mimetic DMOG, as well as the iron chelator 2,2′dipyridyl, decreased hepcidin expression. Moreover, Volke et al. [42] demonstrated that HIF-1*α* or HIF-1*β* knockdown did not increase hepcidin expression and concluded that reductions in hepcidin expression from hypoxia mimetics were not mediated through HIF. Also contrary to previous reports [35, 36, 38, 40, 41], Chaston et al. [43] reported that hypoxia increased Huh7 hepcidin expression. However, when Huh7 cells were cocultured with activated THP-1 macrophages, hypoxia reduced hepcidin expression. Using reporter constructs under control of the hepcidin promoter and the THP-1 coculture model, the investigators demonstrated that hypoxia-induced hepcidin repression in Huh7 cells was abrogated when the SMAD binding site was mutated. Mutations to the Ebox1,2, STAT3, and C/EBP sites had no effect, suggesting that hypoxia-mediated hepcidin regulation is controlled through the SMAD site and is dependent on macrophage coculture. 

SMAD regulation of the hepcidin promoter is mediated through BMPs and bone morphogenetic response elements (BRE) in the hepcidin promoter. Binding of BMPs to their receptors causes phosphorylation of SMAD proteins, binding to the BRE and increased gene expression. The gene product of the HFE2 gene, hemojuvelin, is a coreceptor for BMP proteins, and overexpression increases hepcidin expression in liver cells [17]. Regulation of hepcidin through BMP is independent of inflammatory signaling through STAT3, thus hepcidin may be regulated independently by different stimuli. It has been hypothesized that regulation of hepcidin by hemojuvelin is dependent upon whether hemojuvelin is membrane anchored or soluble. Membrane-anchored hemojuvelin is a positive regulator of the BMP/SMAD cascade, whereas soluble hemojuvelin functions as a dummy receptor, inhibits the pathway, and results in reduced hepcidin expression [44].

Several studies have provided evidence that hepcidin may be regulated by hypoxia through the hemojuvelin/BMP axis and hypoxic regulation of the hemojuvelin cleaving proteins matripase-2 (TMPRSS6) and furin. Du et al. [45] first reported that mice with a homozygous TMPRSS6 mutation, referred to as *mask,* have compromised iron status due to excessive hepcidin signaling. Moreover, hepcidin promoter activation by cotransfection of a hemojuvelin expression construct or recombinant BMP2, BMP4, or BMP9 proteins was blunted when cells were treated with a wild-type TMPRSS6 expression vector but not with a vector coding for TMPRSS6 with the *mask *mutation. Folgueras et al. [46] and Finberg et al. [47] further demonstrated the importance of TMPRSS6 on hepcidin regulation by demonstrating that TMPRSS6 knockout mice have elevated hepcidin levels and decreased iron stores compared to wild-type mice. Furthermore, TMPRSS6 mutations have been described in humans and result in aberrant hepcidin signaling and iron-refractory iron-deficiency anemia [48–51]. TMPRSS6 was demonstrated by Silvestri et al. [52] to cleave membrane hemojuvelin and inhibit hepcidin expression. The same research group [53] also reported that hemojuvelin can be cleaved by furin in the endoplasmic reticulum. Furin promoter activity was increased by the HIF stabilizers DFO and CoCl_2_ and decreased by ferric ammonium citrate treatment suggesting HIF regulation. This is supported by an earlier study demonstrating that furin expression is positively regulated by hypoxia via the HIF pathway [54]. 

Recently, Lakhal et al. [55] demonstrated that TMPRSS6 is regulated by hypoxia through HIF-1*α* and 2*α*. TMPRSS6 gene expression increased in Hep3B cells when cultured at 0.5% O_2_ and in the presence of the hypoxia mimetic DMOG. Increased TMPRSS6 expression induced by hypoxia or hypoxia mimetics was curtailed by siRNA knockdown of HIF-1*α* and/or 2*α*. It was also reported that hypoxia reduced membrane hemojuvelin and this effect could be abrogated by siRNA knockdown of TMPRSS6, HIF-1*α*, and 2*α*. Hypoxia decreased activity of luciferase constructs containing the proximal hepcidin BRE, and this effect was inhibited when cells were treated with either TMPRSS6 or HIF-1*α* and 2*α* siRNA or if the promoter BRE was mutated.

## 5. Localized Adipose Hypoxia and Hepcidin Expression

Early studies have demonstrated a link between obesity and poor iron status [56, 57]. Studies have demonstrated this association in children and adolescents [57–60], adult men and women [61–64], and postmenopausal women [65]. Obesity has been suggested as an independent factor contributing to iron deficiency [60, 61, 63, 65], and studies have correlated obesity, BMI, and increased hepcidin in premenopausal women [66] and children [67]. Chronic inflammation is associated with central obesity and has been implicated in many obesity-related problems such as insulin resistance [68, 69]. Obese mice (ob/ob line) have significantly higher plasma IL-6 levels than their lean counterparts, and this difference is attributed to increased IL-6 expression in adipose tissue, suggesting that adipose tissue contributes to plasma inflammatory cytokine levels [70].

Adipose hypoxia is a contributing factor to increased inflammation associated with obesity. Ye et al. [71] demonstrated that adipose tissue in ob/ob mice is considerably more hypoxic than adipose tissue from lean mice. Differences in O_2_ tension in ob/ob mice increased the expression of the inflammatory cytokines TNF-*α*, IL-1, and IL-6. Increased expression of cytokines was also demonstrated in 3T3-L1 cells cultured under hypoxic conditions (1% O_2_), suggesting that the phenomenon of obesity-induced inflammation may result from increased secretion of inflammatory cytokines from hypoxic adipocytes found in deep fat pockets. Another consequence of increased adiposity and adipocyte hypoxia is macrophage infiltration of adipose tissue. Low partial pressure of O_2_ in human adipose tissue is correlated to macrophage infiltration [72, 73]. Several studies have demonstrated that obesity in humans increases markers of adipose macrophage content and subsequent inflammation [73, 74].

Hepcidin expression is well known to be induced by inflammation. IL-6 mediates hepcidin induction by increasing the binding of the transcription factor STAT3 to hepcidin promoter [20–22]. Considering that central obesity and resulting adipocyte hypoxia increase systemic IL-6 [71], it stands to reason that adipocyte hypoxia may be involved in the hypoferremia of obesity through increased inflammatory signaling of hepcidin. Hintze et al. [75] recently demonstrated that Huh7 hepatocytes treated with conditioned media from 3T3-L1 adipocytes cultured at 1% O_2_ had significantly greater hepcidin promoter activity and mRNA levels compared to Huh7 cells treated with conditioned media from 3T3-L1 cultured at 21% O_2_. Hypoxic adipocytes had increased levels of both IL-6 and leptin mRNA. Leptin and IL-6 have been demonstrated to induce hepcidin expression via the STAT3 pathway [75, 76] suggesting a possible mechanism whereby adipocyte hypoxia may signal hepcidin. When the hepcidin STAT3 binding site was mutated in the hepcidin promoter, activity was diminished, suggesting that adipocyte hypoxia may increase hepcidin expression through increased inflammation.

## 6. Summary

**Figure 2:**
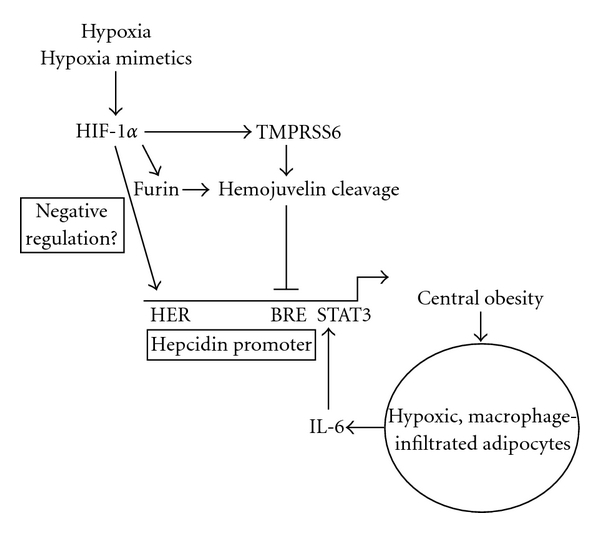
Pathways for hypoxia-mediated hepcidin regulation. Hypoxia or hypoxia mimetics result in HIF-1*α* accumulation. HIF-1*α* has been proposed to be a direct, negative regulator of hepcidin by binding to putative HIF response elements (HRE) in the hepcidin promoter [38]. Expression of the hemojuvelin cleaving enzymes TMPRSS6 and furin is increased by hypoxia via HIF-1*α* [53, 55]. Hemojuvelin cleavage results in decreased hepcidin promoter activation through inhibition of BMP/bone morphogenetic response element (BRE) signaling [44]. Localized, adipocyte hypoxia caused by central obesity results in elevated IL-6 levels which can increase hepatocyte hepcidin expression via the STAT3 binding site [75].

The recent discovery of hepcidin, which has been described as a central regulator of iron homeostasis, has provided evidence that iron balance is a tightly regulated process affected by a series of factors other than diet, to include hypoxia. Hypoxia has profound effects on iron absorption [30–32] and results in increased erythropoiesis when humans move from sea level to altitude [33]. Several mechanisms or combinations thereof may explain decreased hepcidin expression mediated by hypoxia (Figure 2). There is evidence that the hepcidin promoter may be a target for direct negative regulation by the HIF/HRE system [38], although not all studies support this assertion [42]. Despite sometimes conflicting results, studies utilizing hypoxia mimetics that stabilize HIF proteins have consistently demonstrated decreased hepcidin expression [38, 40, 42, 55] suggesting that the HIF system is involved in hepcidin regulation either directly or through upstream mediators. Localized adipose hypoxia due to obesity may in part play a role in hepcidin signaling and organismal iron metabolism. The emerging literature concerning obesity, adipocyte hypoxia, and inflammation suggests that the etiology of obesity-related hypoferremia may be mediated in part through increased hepcidin expression through inflammatory pathways originating from adipose tissue. Research in the area of hypoxia and iron metabolism continues to provide novel evidence of the molecular regulation of hepcidin and its effects on iron status. It is likely that recent findings will result in the development of novel interventions affecting hepcidin expression, and, subsequently, iron balance.
